# Fine mapping and identification of a candidate gene for a major locus controlling maturity date in peach

**DOI:** 10.1186/1471-2229-13-166

**Published:** 2013-10-22

**Authors:** Raul Pirona, Iban Eduardo, Igor Pacheco, Cassia Da Silva Linge, Mara Miculan, Ignazio Verde, Stefano Tartarini, Luca Dondini, Giorgio Pea, Daniele Bassi, Laura Rossini

**Affiliations:** 1 Plant Genomics Section, Parco Tecnologico Padano, Lodi 26900, Italy; 2 Università degli Studi di Milano, DiSAA, Via Celoria 2, Milan 20133, Italy; 3 Istituto di Genomica Applicata, Via Linussio 51, Udine 33100, Italy; 4 Dipartimento di Scienze Agrarie e Ambientali, Università degli Studi di Udine, Via delle Scienze 206, Udine 33100, Italy; 5 Consiglio per la Ricerca e la Sperimentazione in Agricoltura, Centro di Ricerca per la Frutticoltura, Via Fioranello 52, 00134 Roma, Italy; 6 DCA, Department of Fruit Tree and Woody Plant Science, University of Bologna, Bologna 40126, Italy

**Keywords:** Maturity date, *Prunus persica*, QTL, Candidate gene

## Abstract

**Background:**

Maturity date (MD) is a crucial factor for marketing of fresh fruit, especially those with limited shelf-life such as peach (*Prunus persica* L. Batsch): selection of several cultivars with differing MD would be advantageous to cover and extend the marketing season. Aims of this work were the fine mapping and identification of candidate genes for the major maturity date locus previously identified on peach linkage group 4. To improve genetic resolution of the target locus two F_2_ populations derived from the crosses Contender x Ambra (CxA, 306 individuals) and PI91459 (NJ Weeping) x Bounty (WxBy, 103 individuals) were genotyped with the Sequenom and 9K Illumina Peach Chip SNP platforms, respectively.

**Results:**

Recombinant individuals from the WxBy F_2_ population allowed the localisation of maturity date locus to a 220 kb region of the peach genome. Among the 25 annotated genes within this interval, functional classification identified ppa007577m and ppa008301m as the most likely candidates, both encoding transcription factors of the NAC (NAM/ATAF1, 2/CUC2) family. Re-sequencing of the four parents and comparison with the reference genome sequence uncovered a deletion of 232 bp in the upstream region of ppa007577m that is homozygous in NJ Weeping and heterozygous in Ambra, Bounty and the WxBy F_1_ parent. However, this variation did not segregate in the CxA F_2_ population being the CxA F_1_ parent homozygous for the reference allele. The second gene was thus examined as a candidate for maturity date. Re-sequencing of ppa008301m, showed an in-frame insertion of 9 bp in the last exon that co-segregated with the maturity date locus in both CxA and WxBy F_2_ populations.

**Conclusions:**

Using two different segregating populations, the map position of the maturity date locus was refined from 3.56 Mb to 220 kb. A sequence variant in the NAC gene ppa008301m was shown to co-segregate with the maturity date locus, suggesting this gene as a candidate controlling ripening time in peach. If confirmed on other genetic materials, this variant may be used for marker-assisted breeding of new cultivars with differing maturity date.

## Background

Fruit ripening is a complex process that involves the coordinated regulation of many metabolic pathways which influence numerous traits such as colour, aroma and flavour. During ripening, softening of the fruit tissues occurs in parallel with the accumulation of sugars, acids, and volatile compounds. Together, these traits contribute to increased palatability. Other characters, such as colour, size, and maturity date, have been selected to offer products with improved consumer appreciation. For practical reasons, fruits are harvested before they are physiologically ripe and are thus often perceived by the consumer as of poor quality. Consequently, the ideotype pursued by modern peach breeding is characterized by an increase of fruit quality associated to an easy harvest (fruit with a firmer texture or very slow softening), in order to reach optimal fruit quality at consumption. Fruit maturity is an important trait and early fruit ripening could allow market growth by extending the length of the production season. Studies in a wide range of plant species have provided insights into the genetic mechanisms that mediate fruit ripening-related processes, such as pigment synthesis, cell wall and sugar metabolism [1-4].

Climacteric fruits such as peach are characterized by an increase in respiration and an autocatalytic burst of ethylene production late in fruit development which is essential for normal fruit ripening. Blocking ethylene synthesis or perception prevents ripening [5-7]. Some tomato mutants (e.g. *rin*, *nor*, *cnr*) are unable to ripen even when treated with exogenous ethylene, although not impaired in the hormone signal transduction pathway [8]; and references therein]. Molecular analyses of these mutants and identification of the corresponding genes indicate that they are necessary for the expression of both ethylene-dependent and -independent genes during ripening, acting upstream of and possibly in parallel to ethylene [8]. In banana, another climacteric fruit, NAC transcription factors have been shown to physically interact with a downstream component of ethylene signaling, ethylene insensitive 3 (EIN3)-like protein, which was down-regulated during ripening [9]. Thus, the control of NAC TFs on ripening is important as that regulated by the hormone.

In peach, QTLs controlling MD have been mapped on different chromosomes, with major QTLs located on linkage group (LG) 4 and 6 [10-13]. By analyzing the Contender x Ambra (CxA) F_2_ population we have shown that the QTL detected on LG4 behaves as a Mendelian trait and has pleiotropic effects masking the effects of QTLs for different fruit traits [10]. In the present work we refer to this locus as *qMD4.1.* Recently, a major QTL was also identified in the collinear region of apricot (*P. armeniaca* L.) and sweet cherry (*P. avium* L.) suggesting that a common mechanism may control fruit maturation in related *Prunus* species [13].

In the present study, *qMD4.1* was genetically dissected to gain insight into the mechanisms controlling MD in peach and to better understand the genetic interactions of this locus with other fruit traits. To this end, a fine map of the *qMD4.1* locus was constructed, increasing the number of markers and individuals in the CxA population and analyzing another F_2_ population where MD is segregating as a Mendelian trait. Analysis of the region located candidate genes for the MD QTL and a scan of publicly available re-sequencing data for the CxA F_1_ parent identified polymorphisms in the region harbouring *qMD4.1*[14]. A sequence variant in a NAC candidate gene was shown to co-segregate with the MD trait in these two populations and can be used in selection for early/late maturity genotypes.

## Results and discussion

### Fine mapping of the *qMD4.1* locus

The objective of this study was to fine map *qMD4.1*, the MD locus described by Eduardo *et al*. [10] on LG4 of the CxA F_2_ population. This locus had been mapped using 169 individuals and a genetic map comprising 31 SSRs and two phenotypic markers, with flanking markers M12a and EPPISF032 defining a 6.6 cM interval, corresponding to 3.56 Mb on the peach reference genome. In order to fine map *qMD4.1*, the number of individuals of this population was increased to 306 and 14 SNPs were added around and into the original map interval. The SNPs were manually selected from the publicly available genome re-sequencing data of the CxA F_1_ self-pollinated parent (NCBI Sequence Read Archive, biosample SRS335631). In parallel, we also analysed another F_2_ population of 103 individuals derived from the cross between PI91459 (N.J. Weeping; W) and Bounty (By), also segregating for MD and genotyped with the 9K SNP peach array v1 [15] (Da Silva *et al*., unpublished).

**Figure 1:**
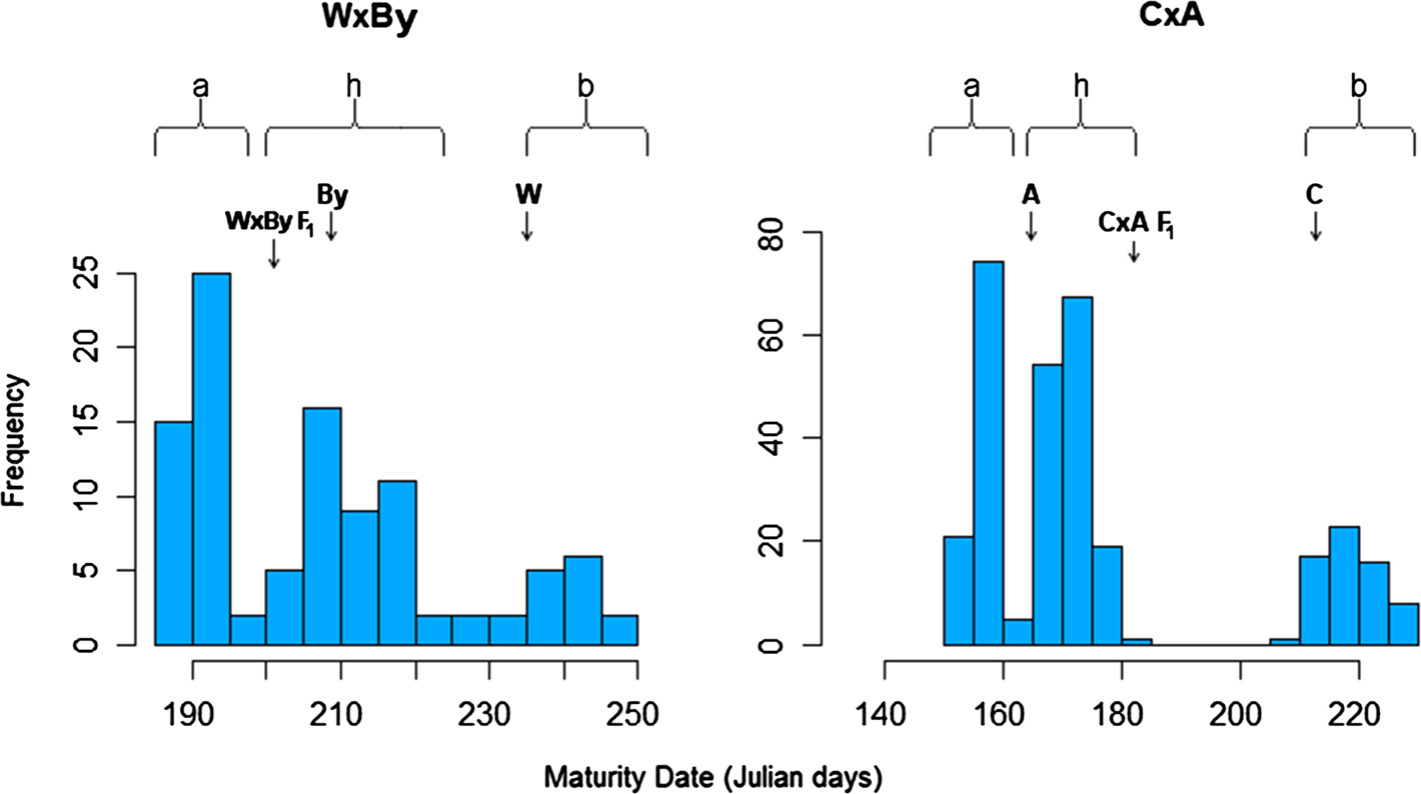
**Trimodal distribution of peach MD trait in the WxBy (left) and CxA (right) populations.** Arrows indicate the position of values detected for the four parents and the two F_1_s. For each cross, we refer to the F_2_ individuals falling in class “**a**”, **h**” and “**b**” as early, intermediate and late ripening, respectively. Each peak of the distribution was considered to represent a genotype for a co-dominant marker in these populations.

In both populations MD showed a trimodal distribution (Figure 1) and very high correlation between years (0.92) (Table 1). This distribution suggested a Mendelian behaviour. Thus, a genome-wide QTL analysis for MD was carried out for confirmation using both populations independently, with the purpose of excluding the possible segregation of other MD QTLs (data not shown). Although segregating as a Mendelian trait in both the CxA and the WxBy F_2_ populations, MD behaves as a quantitative trait in other populations such as the F_1_ population derived from the cross between Bolero and OroA [10].

**Table 1:** **Correlation coefficient, mean, minimum and maximum values, and standard deviation (SD) of maturity date in the CxA and WxBy F**_**2 **_**populations for two successive years respectively**

**Population**	**Years**	**Correlation**	**Mean**	**Min**	**Max**	**SD**
CxA	2007/2008	0.92	176.00/177.70	151.00/156.00	232.00/230.00	23.42/22.86
WxBy	2010/2011	0.92	207.12/205.83	183.00/194.00	250.00/242.00	17.32/13.51

Maturity date is expressed in Julian days at harvest.

**Figure 2:**
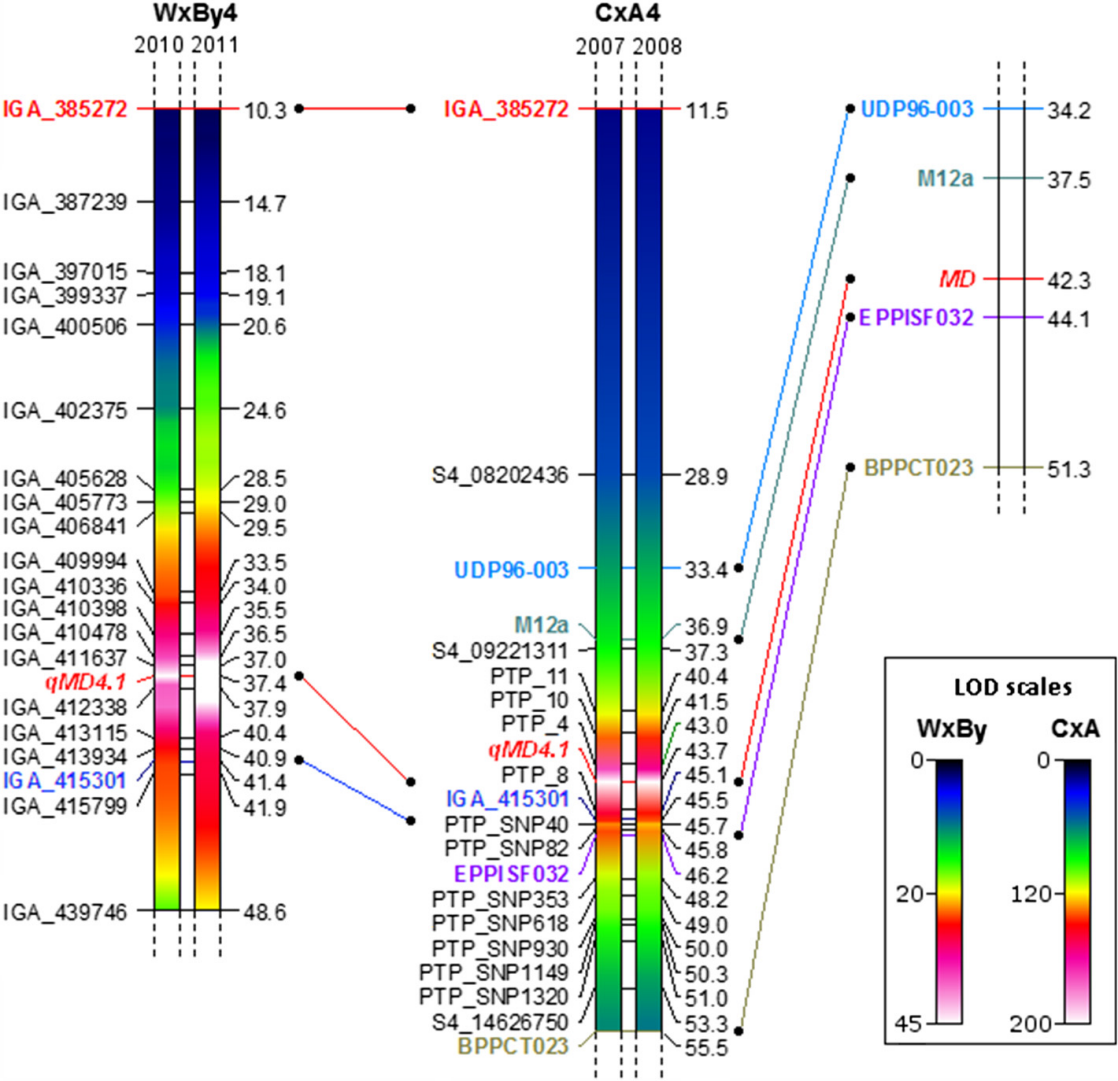
**Map comparison of the linkage groups 4 among WxBy, CxA, and the published SSR map of CxA produced by Eduardo*et al*. [**[10]**].** Coloured lines represent common markers between WxBy and CxA (SNPs) and the CxA of this study with the CxA map of Eduardo *et al*. Location of *qMD4.1* on the delimited region of chromosome 4 (dashed lines at top and bottom) for two successive years in the two studied populations (years 2010 and 2011 for WxBy; years 2007 and 2008 for CxA) as determined by interval mapping. Marker names are listed on the left side and genetic distances (cM) on the right. QTLs are represented as colour gradients corresponding to the respective LOD scales (reported at bottom-right) and drawn with Harry Plotter software (http://genomics.research.iasma.it/download.html).

Figure 2 shows the region where *qMD4.1* is located in both maps, CxA and WxBy, as compared to the data from Eduardo *et al*. [10]. In both maps high segregation distortion was observed in the target region (Table 2). The analysis of the alleles showed that under-represented alleles came from the C genome in CxA and from W genome in WxBy, in both cases corresponding to the late ripening allele.

**Table 2:** LOD scores and percentages of phenotypic variation in CxA and WxBy QTL analyses

**CxA**				**WxBy**			
**Position (cM)**	**Locus**	**LOD**	**% Expl.**	**Position (cM)**	**Locus (c)**	**LOD 2010/2011**	**% Expl. 2010/2011**
		**2007/2008**	**2007/2008**				
33.443	UDP96003^a^*	65.3/67.5	64.4/65.3	34.015	SNP_IGA_410336^c^****	29.59/32.48	78.7/77.6
34.443		71.7/74.1	67.8/68.6	34.015	SNP_IGA_410265^c^****	29.59/32.48	78.7/77.6
35.443		76.1/78.6	70.0/70.8	35.015		36.24/37.43	85/82.2
36.443		77.8/80.3	70.8/71.6	35.493	SNP_IGA_410398^c^****	37.92/38.45	86.3/83
36.909	M12a^a^*	77.6/80.1	70.7/71.5	36.473	SNP_IGA_410478^c^****	41.47/43.07	88.6/86.2
37.301	S4_09221311^c^**	80.3/83.5	71.9/73.0	36.961	SNP_IGA_411637^c^****	43.15/45.41	89.5/87.6
38.301		97.7/102.3	78.7/79.8	**37.449**	**MD**^a^****	**47.81/52.85**	**91.8/91.2**
39.301		111.5/117.3	82.9/84.1	37.937	SNP_IGA_412338^c^*****	41.87/49.34	88.8/89.7
40.301		117.6/123.7	84.5/85.6	38.937		42.12/48.2	89/89.1
40.358	PTP_11^b^**	117.7/123.8	84.5/85.6	39.937		37.29/41.04	85.8/84.9
41.358		144.0/152.9	89.8/90.9	40.425	SNP_IGA_413115^c^******	34.16/37.05	83.3/81.8
41.522	PTP_10^b^**	144.8/153.5	89.9/91.0	40.912	SNP_IGA_414017^c^******	31/35.93	80.3/80.9
42.522		179.7/176.5	94.2/93.7	41.400	SNP_IGA_415301^c^*****	28.58/34.91	77.6/80
43.023	PTP_4^b^**	185.7/178.6	94.7/93.9	41.888	SNP_IGA_415799^c^****	27.32/33.99	76.1/79.1
**43.681**	**MD**^a^**	**231.2/224.1**	**97.4/97.0**				
44.681		194.1/183.1	95.4/94.3				
45.149	PTP_8^b^**	166.2/157.4	92.8/91.5				
45.507	SNP_IGA_415301^c^*	154.5/146.3	91.3/89.9				
45.671	PTP_SNP40^b^**	146.0/137.6	90.1/88.4				
45.834	PTP_SNP82^b^**	139.3/131.1	89.0/87.2				
46.193	EPPISF032^a^*	148.6/139.5	90.5/88.8				

The highest LOD and percentage of phenotypic variation are indicated in bold with the nearest marker. Positions are indicated in cM (centiMorgan). (a) Markers from Eduardo *et al*. [10], (b) newly developed markers, (c) markers from Verde *et al*. [17]. Significance levels of locus-genotype frequencies: *:0.1 **:0.05 ***:0.01 ****:0.005 *****:0.001 ******:0.0005 *******:0.0001.

To define the target region for candidate gene analysis and to anchor the two maps we used the Sequenom platform to genotype the CxA F_2_ progeny for 2 SNPs that were also included in the 9K SNP peach array v1 (i.e., SNP_IGA_385272 and SNP_IGA_415301; Figure 2).

Considering the region between the anchor markers SNP_IGA_385272 and SNP_IGA_415301 present in both populations, in the CxA map, the region spans 33.99 cM and includes 2 SSRs and 6 SNPs, whereas in the WxBy fine map this region includes 16 SNPs and spans 31.12 cM. The full map from WxBy will be described elsewhere and was used here to run a QTL analysis for the MD trait, showing no other QTL for MD segregating in this population (data not shown). Fine mapping analysis showed that in CxA, *qMD4.1* is localized in a window of 2.13 cM between the markers PTP4 and PTP8 spanning the chromosome region between 10.87-12.09 Mb, whereas in the WxBy map *qMD4.1* is located in a window of 0.98 cM between markers SNP_IGA_411637 and SNP_IGA_412338 spanning the chromosome region between 10.97-11.19 Mb (Table 2). Moreover, in the CxA progeny LOD score in *qMD4.1* region reaches a value of more than 220 (97% of explained variance) while in WxBy LOD score is about 50 with more than 90% of explained variance (Table 2). Therefore this is the region where we focused the search for candidate genes.

**Figure 3:**
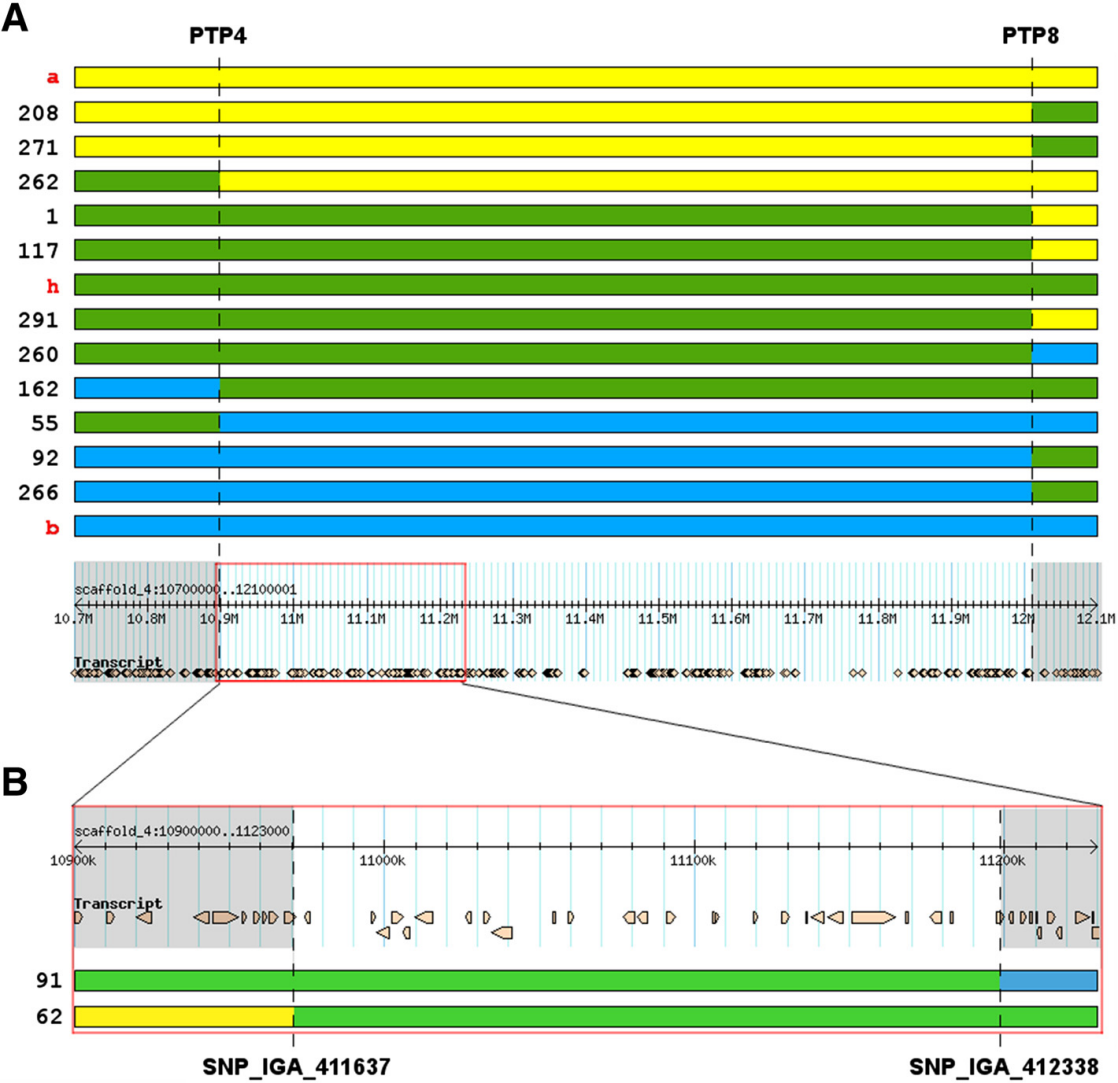
**Graphical representation of the region of the*qMD4.1*locus delimited by recombinants analysis. A)** Recombinants identified in the CxA population delimiting the region between SNP markers PTP4 and PTP8 (dashed lines) and aligned to the reference genome (bottom). The red rectangle on the reference genome panel is the region delimited by the WxBy recombinants. **B)** Detailed region of the *qMD4.1* delimited by the SNP marker SNP_IGA_411637 and SNP_IGA_412338 in the WxBy population and aligned to the reference genome (top). The numbers on the left in **A)** and **B)** represent the accession ID of the F_2_ individuals, whereas **a**, **h**, and **b** in red colour represent the three ideal haplotypes. The brownish objects in **A)** and **B)** under the “transcript” headings on the reference genome panels represent the annotated genes.

Although the CxA F_2_ population has a higher number of individuals, the WxBy F_2_ population has a higher number of segregating genotyped markers, which provided a better resolution in the mapping of *qMD4.1* (Figure 3). These results showed the utility of the new genomic tools, such as the 9K SNP peach array v1 [15], to improve the efficiency and resolution in peach mapping studies. In addition, the use of the WxBy F_2_ population allowed us to identify a relatively small region harbouring a limited number of candidate genes compared to the CxA F_2_ population (Figure 3).

QTLs for MD in a region overlapping with *qMD4.1* were already identified by other groups [11,16]. For example, Dirlewanger *et al*. [13] mapped a major QTL for MD on LG4 using an F_2_ population derived from the cross Ferjalou Jalousia x Fantasia (JxF): based on available data, *qMD4.1* seems to overlap with their confidence intervals in different years, as supported by the SSRs UDP97-402 and AMPA 103, that in JxF defined this region and are located on the peach genome sequence in scaffold 4 at position 10,486,180 and 13,509,355, respectively (approximately 3.02 Mb apart). In contrast to the Mendelian behaviour of *qMD4.1* in our populations, in these previous studies MD segregated as a quantitative trait with QTLs mapped on different chromosomes. Interestingly, these studies showed that the region spanning *qMD4.1* hosted a cluster of QTLs associated with other traits such as fructose and sucrose content, titrable acidity, pH, sorbitol, maltose, citric acid, and quinic acid content [11]. Recently, an association mapping study showed that another locus mapped at the beginning of chromosome 4, associated with SSR marker CPPCT028 (at 2.1 Mb) was strongly associated with variation for the MD trait [17]. This indicates that other loci controlling MD can be identified using different segregating populations or association mapping. In addition, strong associations between MD, SSC, flavonoids, sorbitol, and total sugars levels were also found in this study [17].

### Identification and analysis of candidate genes

Based on overlapping map positions of the *qMD4.1* locus in the CxA and WxBy F_2_ populations, our hypothesis was that allelic variation at the same gene could explain the segregation of the MD trait in both populations. Thus, we searched for genomic variations within the region 10.97-11.19 Mb identified by two WxBy recombinants (Figure 3). Twenty-five genes are annotated in this region of the peach genome (Table 3) and we focused our attention on ppa007577m and ppa008301m, both predicted to encode NAC transcription factors (TFs). NACs constitute one of the largest plant TF families and are key regulators of developmental programs and stress response [18-20], and references therein]. Initially, we Sanger re-sequenced ppa007577m in the four parents C, A, W and By trying to identify polymorphisms within the gene, as well as in the 1.5 kb upstream and downstream flanking sequences. Sequence analysis did not show polymorphisms within the coding region, but revealed a 232 bp deletion, compared to the reference genome (Lovell), at 579 bp upstream of the start codon. PCR amplification of this region showed that C is homozygous for the reference Lovell allele, W homozygous for the deletion, whereas A and By are both heterozygous (data not shown). In addition, PCR analysis of the CxA F_1_ parent for the 232 bp deletion showed that this individual was homozygous for the reference Lovell allele (C haplotype). Hence, no segregation was expected in the F_2_ CxA population. To further verify this hypothesis, inspection of this deletion was conducted screening some individuals of the three phenotypic classes (early, middle and late ripening) of the CxA F_2_ population. As expected from the parents’ analysis, the deletion of 232 bp was absent in the analysed individuals. In contrast, the 232 bp deletion co-segregates with the phenotype in the selected individuals of the WxBy F_2_ population being the F_1_ parent heterozygous at this locus. Altogether, these results led us to search for others candidate genes for the MD trait because ppa007577m does not segregate with the MD trait in both populations.

**Table 3:** **Annotated genes found in the *qMD4.1 *region restricted by the WxBy recombinants**

**Peach gene id**	**Transcript start**	**Transcript stop**	**Description**	**Pathway**
ppa022868m	10974197	10975795	CBS domain-containing protein	Response to wounding
ppa010307m	10995538	10997054	Stem-specific protein TSJT1	Anther-specific gene
ppa008860m	10997481	11001496	Ribosome biogenesis protein BRX1 homolog	Ribosome biogenesis
ppa006721m	11002389	11005897	LanC-like protein 2	Regulation of abscisic acid mediated signaling
ppa006788m	11005900	11008016	Protochlorophyllide reductase	Chlorophyll biosynthetic process
ppa001338m	11009697	11015503	WD repeat-containing protein 44	Endosome membrane (in animal)
ppa012067m	11026020	11028069	Unknown protein	Unknown
ppa019135m	11031823	11034176	Unknown protein	Unknown
ppa000375m	11034671	11041074	CIP7 (COP1-INTERACTING PROTEIN 7)	Anthocyanin and chlorophyll accumulation
ppa017913m	11054156	11055142	TMV resistance protein N	Pathogen response
ppa008896m	11059223	11060983	Peroxidase 47	Response to oxidative stress
ppa025660m	11076787	11080515	Beta-glucosidase 18	Lignin biosynthetic process
ppa017816m	11081663	11084616	Beta-glucosidase 18	Lignin biosynthetic process
ppa014861m	11090833	11093934	ABC transporter G family member 9	Transport
ppa008301m	11105983	11107728	NAC domain-containing protein 72	Transcription factor
ppa021959m	11119202	11120235	Unknown protein	Putative transposon
ppa007577m	11128296	11130419	NAC domain-containing protein 18	Transcription factor
ppa023456m	11135882	11136154	Unknown protein	Putative transposon
ppa007209m	11137776	11141711	Peroxisomal membrane protein 2	Peroxisomal membrane pore-forming activity
ppa005409m	11143135	11148005	“Peptidyl-prolyl cis-trans isomerase CYP37	Protein folding
ppa000348m	11150940	11164717	Trafficking protein particle complex subunit 10	Transport
ppa009230m	11168222	11169155	Methyltransferase	Unclear
ppa007114m	11176100	11179631	PAP-specific phosphatase HAL2-like	Sulfur compound metabolic process
ppa014087m	11182744	11183404	Unknown protein	Unknown
ppa001400m	11197319	11200080	Receptor-like protein kinase THESEUS 1	Unidimensional cell growth

**Figure 4:**
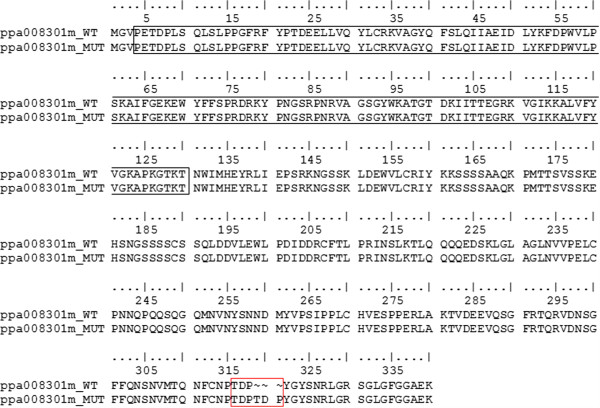
**Sequence alignment of the two variants of the NAC TF ppa008301m.** ppa008301m_WT indicates the protein sequence without the deletion (identical to the reference genome) whereas ppa008301m_MUT indicates the three amino acids insertion found in this study (red box). The black box indicates the NAC domain.

We thus focused our attention on the second NAC candidate gene ppa008301m. The Sanger re-sequencing of the four parents and of the two F_1_ individuals uncovered an in-frame 9 bp insertion (compared to the reference genome) resulting in a tandem duplication of three amino acids in the C-terminal domain (Figure 4). A, C, and By are heterozygous for this variant, whereas W is homozygous without the 9 bp insertion. Both CxA and WxBy F_1_ individuals were heterozygous for this polymorphism and further analyses on the F_2_ individuals were conducted to verify its correct segregation with the MD trait. To this end, the 9 bp insertion/deletion (INDEL) was scored using the genotyping protocol used for microsatellites analyses (data not shown). The allelic variants co-segregated with the MD of all the individuals in both F_2_ populations, with the early ripening individuals showing the 9 bp insertion and the late ripening individuals carrying the Lovell reference allele (Additional file 1). To further verify the map position, we added these data to the previous genotyping dataset used for map construction and QTL analysis. We found that this INDEL co-mapped with the MD phenotypic marker (Additional file 2). Finally, repeating the QTL analysis in both populations showed co-location of this INDEL with *qMD4.1* (Additional file 2).

As an additional confirmation that this 9 bp INDEL found in the last exon of the NAC gene ppa008301m is the most probable polymorphism within the *qMD4.1* region, we searched the publicly available paired-end whole-genome next-generation sequencing data of the CxA F_1_ parent for sequence variants within the original target region. Table 4 reports the results of this analysis. Most of the identified polymorphisms were located in intergenic regions. Only two polymorphisms were found within or near coding regions: the first in the 5′-UTR of a β-glucosidase 18 gene (ppa025660m) involved in carbohydrate and phenylpropanoid metabolism, and the second being the 9 bp insertion in the NAC candidate gene ppa008301m. Even if polymorphisms in intergenic regions could cis-regulate the expression of target genes, the presence of the 9 bp insertion in the coding sequence of this NAC candidate gene supports the hypothesis that the NAC TF ppa008301m is a strong candidate gene for the control of MD in the CxA and WxBy populations.

**Table 4:** **Detection of polymorphisms in CxA F**_**1 **_**parent from whole-genome re-sequencing data in the *qMD4.1 *region delimited by the WxBy recombinant individuals**

**Position (bp)**	**Polymorphism type (bp)**	**Polymorphism position**	**Peach transcript id**	**Annotation**
10,995,012	SNP	Intergenic		
10,995,076	INDEL (2)	Intergenic		
11,041,083	INDEL (2)	Intergenic		
11,076,551	INDEL (1)	5*'* UTR	ppa025660m	Predicted β-glucosidase 18
11,086,901	SNP	Intergenic		
11,097,272	SNP	Intergenic		
11,107,278	INDEL (9)	In frame insertion	ppa008301m	NAC domain-containing protein 72
11,115,079	INDEL (2)	Intergenic		
11,115,085	INDEL (8)	Intergenic		
11,115,087	INDEL (3)	Intergenic		
11,115,946	INDEL (7)	Intergenic		
11,115,948	SNP	Intergenic		
11,115,953	INDEL (6)	Intergenic		
11,115,961	INDEL (3)	Intergenic		
11,115,968	INDEL (3)	Intergenic		
11,116,073	INDEL (15)	Intergenic		
11,116,145	INDEL (59)	Intergenic		
11,116,147	SNP	Intergenic		
11,142,067	INDEL (1)	Intergenic		
11,142,748	INDEL (1)	Intergenic		
11,190,195	SNP	Intergenic		

**Figure 5:**
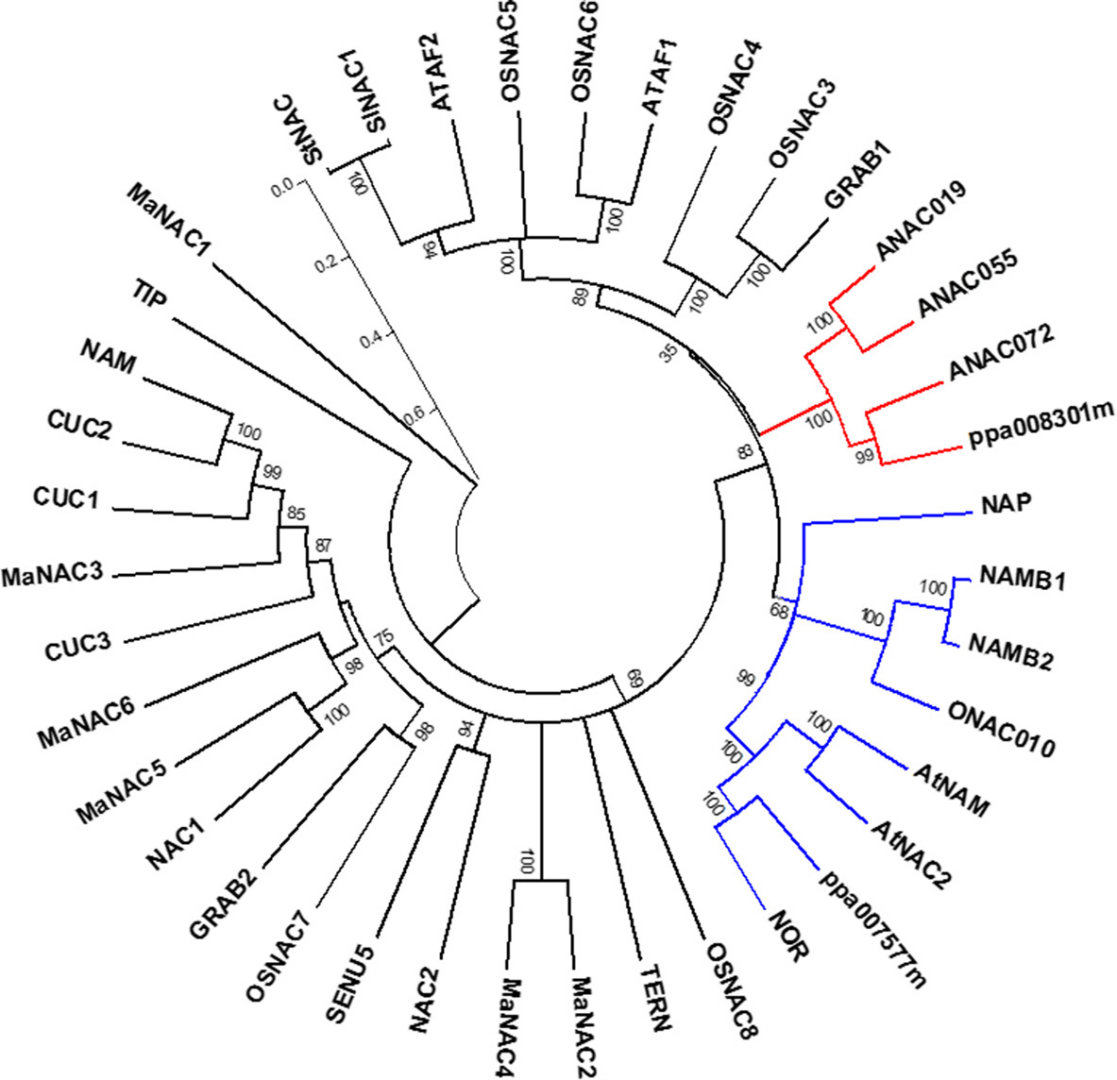
**Phylogenetic analysis of peach NAC TF ppa007577m and ppa008301m with NAC TFs identified in other species.** The gene names and UniProtKB/Swiss-Prot accession numbers were as follows. *Oryza sativa*: OsNAC3, OsNAC4, OsNAC5, OsNAC6, OsNAC7, OsNAC8, ONAC010; *A. thaliana*: ATAF1 and ATAF2, NAP, CUC1, CUC2, CUC3, AtNAC2, NAC1, NAC2, AtNAM, TIP; ANAC019, ANAC055, ANAC072; *Petunia hybrida*: NAM; *Solanum lycopersicum*: NOR, SlNAC1; *Solanum tuberosum*: StNAC1; *Lycopersicon esculentum*: SENU5; *Nicotiana tabacum*: TERN; *Triticum sp*.:GRAB1, GRAB2, NAM-B1, NAM-B2; *Musa acuminata*: MaNAC1, MaNAC2, MaNAC3, MaNAC4, MaNAC5, MaNAC6. Red and blue branches indicate the two clustering groups of ppa008301m and ppa007577m, respectively. Numbers on branches indicate bootstrap values. Thin bar indicates the scale. Accession number of selected protein sequences are indicated in Methods.

A phylogenetic analysis of NACs identified in *Arabidopsis*, rice, wheat, potato, banana and tomato indicates that NAC genes ppa007577m and ppa008301m belong to two distinct clades (Figure 5). NAC ppa007577m clustered with tomato NOR (non-ripening) known to be involved in fruit ripening (GeneBank accession n° AY573802). First mapped on chromosome 10 by Giovannoni *et al*. [21], the *nor* gene seems to have a global effect on fruit ripening, probably acting upstream of ethylene synthesis [22]. Related *Arabidopsis* genes *AtNAC2* and *AtNAM* (namely *NARS1* and *NARS2*, respectively) (Figure 5) control embryogenesis by regulating the development and degeneration of ovule integuments [23]. The clade of ppa007577m includes also rice ONAC10 and wheat NAC TFs NAM_B1 and NAM_B2 (Figure 5). NAM-B1 and NAM-B2 have been shown to be involved in delaying seed ripening with pleiotropic effects on grain protein, zinc, and iron content [24]. In contrast, functional analysis of *ONAC10*, initially selected by the authors as the most similar rice NAC TF to the wheat NAM_B1, excluded a role in seed ripening while showing that it is essential for correct anther development [20]. Altogether, genes from the ppa007577m clade seem to have undergone a functional diversification, playing roles in distinct biological processes. The clade of NAC TF ppa008301m included genes mainly involved in stress responses as *Arabidopsis* ANAC072, ANAC055 and ANAC019 [25]. Recently, it has been shown that the closely related ANAC019 and ANAC055 play different roles during developmental senescence [26]. In fact, ANAC019 may be involved in triggering senescence by controlling and activating flavonoid and anthocyanin biosynthesis, whereas ANAC055 seems to be involved in the response to chitin [26]. In addition, these genes showed opposing roles in the regulation the jasmonic and salicylic acid pathways [26]. Unfortunately, literature about the involvement of NAC TFs in ripening is at present very scanty. In banana gene expression profiles in fruit with four different ripening characteristics revealed that *MaNAC* genes are expressed differentially in peel and pulp during ripening [9]. Additionally, MaNAC1 and MaNAC2 physically interact with MaEIL5 (involved in ethylene signalling), suggesting that these TFs could be involved in banana fruit ripening via interaction with ethylene signaling components.

Our results also support the involvement of ppa008301m in the control of peach fruit ripening, indicating that genes in this clade may have evolved to fulfill different biological functions as proposed for the ppa007577m clade. While orthologs are often assumed to retain equivalent functions in different organisms, deviations from this situation are not uncommon [9,27]. Being NACs one of the largest TF families, indication of the precise function of each member should be experimentally validated. The 9 bp INDEL in candidate gene ppa008301m results in the duplication of a threonine-aspartic acid-proline stretch with possible impact on protein function. Simple amino acid repeats and regions rich in serine and threonine, proline and glutamine, or acidic residues are frequent in the C-terminal region of NAC proteins [28-33]. While few studies were focused on NAC post-translational modifications, lysine 268 and 269 in tomato SlNAC1 C-terminal region contribute additively to its degradation, whereas the substitution of both lysine residues with two aspartic acids significantly stabilized the SlNAC1 C-terminal protein sequence [34]. In addition, the C-terminal regions of several NAC proteins function as transcriptional activation domains [19]; and references therein]. Domain deletion analysis on the *Arabidopsis* NAC gene *ATAF1*, revealed that the transactivation activity was conferred by the C-terminal domain and did not require the N-terminal domain [35]. Hence, the three amino acid INDEL in ppa008301m, possibly modifying the C-terminal domain structure, might influence the protein stability or its transcriptional activation capability and, consequently, the transcriptional activation of target genes.

Interestingly, we found in both populations that the early ripening individuals possessed the three amino acid insertion in the protein sequence, whereas delayed ripening was associated with the reference allele.

## Conclusions

In this study we report the fine mapping of a major locus controlling maturity date trait in peach, using two different populations. The strategy allowed us to restrict the previously described locus from 3.56 Mb to 0.22 Mb. The NAC gene ppa008301m was identified as a strong candidate within this region and a consistent 9 bp insertion in its last exon was proposed as the variant possibly causing early ripening. Further experiments are needed to explore the functional significance of this INDEL. In any case, the marker developed on this sequence polymorphism provides a convenient molecular tool to discriminate early vs. late ripening individuals within the CxA, WxBy and likely other breeding populations. Considering the proposed pleiotropic effect of *qMD4.1* on other important fruit traits, our results provide a basis to better define breeding programs for fruit quality improvement.

## Methods

### Plant material and phenotyping

Two F_2_ populations were used in this study: a population of 306 individuals derived from the cross between Contender (C) and Ambra (A), and a second population of 103 individuals derived from the cross between N.J. Weeping (W) and Bounty (By). Trees were located in the ASTRA orchards in Castel S. Pietro and Tebano (Emilia Romagna, Italy). Trees of CxA and WxBy progenies were planted on their own roots with a spacing of 1 m within and 4 m between rows and trained as slender spindle (one stem with short lateral scaffolds). Pruning was performed yearly and standard cultural practices were applied. The fruits were thinned before pit hardening to a load of only 30–40 fruits per tree according to vigour, in order to allow a full expression of fruit size not limited by competition.

Twenty fruits per tree were harvested at commercial maturity based on visual colour change and manual evaluation of firmness and the date was recorded. Maturity date (MD) phenotyping was obtained in years 2007–2008 and 2010–2011 for CxA and WxBy, respectively. The MD is defined in Julian days at harvest.

### DNA extraction and genotyping

DNA extraction from young leaves was carried out using the DNeasy 96 Plant Kit (QIAGEN). Microsatellite (SSR) amplifications were performed following the multiplex-ready PCR protocol [10].

### SNP selection, design and genotyping of the CxA population

For the CxA F_2_ population, based on the confidence interval identified by Eduardo *et al*. [10], fine mapping was conducted on the 306 individuals with 16 newly developed SNPs spanning the interval 9–12 Mb on LG4. Putative SNPs were manually selected among those available from sequencing of the F_1_ CxA and the relevant surrounding sequences (300 bp) were downloaded from the peach Gbrowse available on the IGA website (http://www.appliedgenomics.org/). The Mass ARRAY Assay Design 3.1 software was used to design multiplex reactions in which the selected SNPs were included. Genotyping was performed using iPLEX Gold technology [36] and Mass ARRAY high-throughput DNA analysis mass spectrometry (Sequenom, Inc). All the 14 selected SNPs gave high quality results and were used for further analyses. Information about the primers used for the genotyping is listed in Additional file 3.

### SNP selection, design and genotyping of the WxBy population

The 103 individuals from the WxBy F_2_ population were genotyped using the recently developed Illumina 9,000 SNP array v1 for peach [15]. For SNP array genotyping, DNA was extracted with the DNeasy 96 Plant kit (Qiagen), diluted to 50 ng/μl and sent to IASMA Research and Innovation Centre (San Michele all’Adige, Italy) for genotyping. Genotyping was performed following the manufacturer’s recommendations as described in Verde *et al*. [15]. SNP data were scored using GenomeStudio Data Analysis software (Illumina Inc.) using a GenCall threshold of 0.15. SNPs with GenTrain score < 0.6 and those showing severe segregation distortion (χ2 test, p < 10^-6^) and more than 1% of missing data were excluded.

### Genetic linkage maps

Genetic linkage analysis and map construction were performed with JoinMap 4 [37]. CxA and WxBy maps were produced and analyzed as “F2” population in the JoinMap 4 software. The recombination threshold value was set at 0.40 and the Kosambi mapping function was used to convert recombination frequencies into map distances. Markers showing distorted segregation were included in the linkage analysis. All linkage groups of both CxA and WxBy maps were calculated at a minimum LOD score of 5. Linkage maps were drawn using the MapChart 2.1 software [38].

### QTLs analysis

QTL analysis was carried out using the software MAPQTL version 6.0 [39]. After a test of 10,000 permutations, LOD thresholds of 3.9 in 2007 and 3.8 in 2008 in CxA map, and 3.6 in 2010 and 3.5 in 2011 in WxBy were assessed to accept QTL significance. A stringent significance level of p = 0.005 was adopted as threshold for the detection of a QTL for the individual test in order to obtain an overall significance level of about p = 0.05, as previously suggested by Van Ooijen [39]. QTLs were drawn using the MapChart 2.1 software [38]. For each plant of both F_2_ populations, maturity date phenotypic data and genotypes of the 9 bp insertion/deletion in the gene model ppa008301m are reported in Additional file 1. In order to visualize the genetic map and QTL features, QTL-LOD profiles were rendered in heatmaps produced by Harry Plotter software developed at the Edmund Mach Foundation (http://genomics.research.iasma.it/download.html).

### Discovery and genotyping of the NAC ppa007577m and ppa008301 9 bp INDELs

Sanger sequencing for the candidate genes ppa007577m and ppa008301m was conducted spanning the region 11,126,755-11,131,088 and 11,105,608-11,107,907, respectively, with several primer combinations as described in Additional file 4. The 232 bp deletion in gene ppa007577m was identified by amplifying with the forward and reverse primers 5′-CTACTCATACCCGCCAAGGA-3′ and AACGTCGTCATGAGGTACCC, respectively. PCR reactions contained 1–20 ng of genomic DNA, 1x PCR reaction buffer (16 mM, (NH_4_)_2_SO_4_, 67 mM Tris-Cl pH 8.8 and 0.1% Tween-20), 3 mM MgCl_2_, 0.2 mM of each dNTP, 40 nM of forward and reverse specific primers (Sigma Life Sciences), 0.1 U of EuroTaq DNA polymerase (EuroClone) and sterile distilled water to 20 μl final volume. The amplification settings consisted in an initial denaturation step of 2 min at 95°C; 30 amplification cycles of 30 s at 94°C, 30 s at 60°C, 30 s at 72°C and a final extension of 5 min at 72°C. PCR products were loaded on a 0.8% agarose gel (Sigma Life Sciences). SSR amplifications were performed following the multiplex-ready PCR protocol as described by Hayden *et al*. with some modifications [40]. The forward NAC ppa008301-INDEL-specific primer used was synthesized adding at the 5′ end the sequence 5′-ACGACGTTGTAAAA-3′. The protocol also included the use of short, generic primers tagF fluorescently labeled with VIC fluorescent dye (5′-ACGACGTTGTAAAA-3′). PCR reactions contained 1–20 ng of genomic DNA, 1x PCR reaction buffer (16 mM, (NH_4_)_2_SO_4_, 67 mM Tris–HCl pH 8.8 and 0.1% Tween-20), 3 mM MgCl_2_, 0.2 mM of each dNTP, 100 nM of each tag primer, 40 nM of NAC-INDEL-specific primers (forward: 5′-AGAACTCAGCGGGTTGATAACT-3′; reverse: 5′-TGCACCCCTACTCGATTTCT-3′; Sigma Life Sciences), 0.01 U of EuroTaq DNA polymerase (EuroClone) and sterile distilled water to 8 μl final volume. The amplification program consisted in an initial denaturation step of 2 min at 95°C; 20 pre-amplification cycles of 30 s at 92°C, 30 s at 60°C, 30 s at 72°C; 40 amplification/labeling cycles of 15 s at 92°C, 30 s at 54°C, 30 s at 72°C; and a final extension of 5 min at 72°C followed by 25 min at 25°C.

### Detection of polymorphisms in CxA F_1_ parent from whole-genome re-sequencing data

Publicly available paired-end whole-genome re-sequencing data of *Prunus persica* accessions (study SRP013437) were downloaded from the NCBI Sequence Read Archive (SRA, http://www.ncbi.nlm.nih.gov/Traces/sra, [41]. For this study we considered biosamples SRS335634 (Lovell Clone PLov2-2 N, run SRR502985), and SRS335631 (F1 Contender x Ambra, run SRR502997). SRA data of each run were dumped in fastq format using the *fastq-dump* tool of the NCBI sratoolkit v.2.1.16 software (http://www.ncbi.nlm.nih.gov/Traces/sra/sra.cgi?view=software); forward and reverse paired reads were split for each sample into two separate files (option *–split-files*). Reads were quality trimmed by Trimmomatic v.0.22 [42], trimming leading and trailing bases below a quality threshold of 20, also removing reads having an average quality below 20 (calculated on 8 bp long sliding windows) and trimmed reads shorter than 24 bp. For each sample, only reads passing the quality filtering as matching pairs were retained and aligned to the whole *Prunus persica* reference genome v1.0 (http://www.rosaceae.org, [14]) using the Burrows-Wheeler Alignment Tool (BWA) v.0.6.2 [43]. The *aln* (IS linear-time algorithm) and *sampe* (all default options except *–n* 25 –*N* 25) commands were applied, respectively, for constructing suffix array (SA) coordinates of good hits of each individual read and to convert them to chromosomal coordinate and pair the reads. The resulting SAM files were converted to sorted BAM files compliant to the Genome Analysis Toolkit (GATK) format by Picard Tools v.1.77 (http://picard.sourceforge.net/) using, in the order, tools *CleanSam*, *SamFormatConverter* and *AddOrReplaceReadGroups*. GATK-compliant BAM files were submitted to GATK v.2.3-3 [44] for pre-processing procedures, consisting of indel realignment, duplicate removal and base quality score recalibration (BQSR). The data table needed for the recalibration step in BQSR was manually generated upon validated SNP data from the Peach 9 K chip array [15]. Variant discovery procedures were then applied using whole-genome recalibrated alignments across both CxA and Lovell sample simultaneously using GATK *HaplotypeCaller* tool applying standard hard filtering parameters [45].

### Phylogenetic analysis

Known NAC family proteins were collected from the literature and from UniProtKB/Swiss-Prot (http://www.uniprot.org) [46]. The gene names and UniProtKB7Swiss-Prot accession numbers were as follows. *Oryza sativa*: OsNAC3 [Q7EZT1], OsNAC4 [Q52QH4], OsNAC5 [Q53NF7], OsNAC6 [Q7F2L3], OsNAC7 [Q5Z6B6], OsNAC8 [Q7GCL7], and ONAC010 [Q8H4S4] [31]; *A. thaliana*: ATAF1 [Q39013] and ATAF2 [Q9C598] [32], NAP [O49255] [47], CUC1 [Q9FRV4] CUC2 [O04017], and AtNAC2 [Q9LD44], [48], CUC3 [Q9S851] [49], NAC1 [Q84TE6] [50], NAC2 [Q84K00] [46], AtNAM [Q9ZNU2] [29], TIP [Q9LKG8] [51], ANAC019 [Q9C932], ANAC055 [Q9LDY8], and ANAC072 [Q93VY3] [52]; *Petunia hybrida*: NAM [Q40880] [32]; *Solanum lycopersicum*: NOR [Q56UP7] [46], SlNAC1 [Q6RH27] [34]; *Solanum tuberosum*: StNAC1 [Q948Z2] [46]; *Lycopersicon esculentum*: SENU5 [Q43521] [53]; *Nicotiana tabacum*: TERN [Q9SXQ0] [46]; *Triticum sp*.: GRAB1[Q9ZRZ3] and GRAB2 [Q9ZRZ2] [33], NAM-B1 [A0SPJ4] and NAM-B2 [A0SPJ6] [24]; *Musa acuminata*: MaNAC1 [M0TAJ5], MaNAC2 [M0TBV3], MaNAC3 [M0U410], MaNAC4 [M0TDM6], MaNAC5 [M0U3M1], MaNAC6 [M0RNB9] [9]. The phylogenetic analysis was inferred using the Neighbor-Joining method [54]. The bootstrap consensus tree inferred from 1000 replicates [55] is taken to represent the evolutionary history of the taxa analysed [55]. Branches corresponding to partitions reproduced in less than 50% bootstrap replicates are collapsed. The evolutionary distances were computed using the Poisson correction method [56] and are expressed as number of amino acid substitutions per site. The analysis involved in total 39 protein sequences, including ppa007577m and ppa008301m. All ambiguous positions were removed for each sequence pair. There were a total of 708 positions in the final dataset. Evolutionary analyses were conducted in MEGA5 [57].

## Competing interests

The authors verify that there are no competing interests.

## Authors’ contributions

RP (designed the experiments; conducted the experiments; analysed data; wrote the manuscript); IE (analysed data; wrote the manuscript); IP (collected and analysed phenotypic and genotypic data; revised the manuscript); CDS (analysed data; revised the manuscript); MM (analysed data); IV (provided re-sequencing data prior to publication); ST (genotyping, analysed data; revised the manuscript); LD (genotyping; analysed data; revised the manuscript); GP (analysed data); DB (selected the genetic materials and developed the populations; revised the manuscript); LR (conceived and designed the study; analysed data; helped writing the manuscript). All authors read and approved the final manuscript.

## Supplementary Material

Additional file 1 **Tables reporting the maturity date and the corresponding genotype of both CxA and WxBy F**_**2 **_**individuals analysed in this study.** Click here for file

Additional file 2 **Repeated QTL analysis including genotyping data from the 9 bp INDEL found in the ppa008301m candidate gene (INDEL).** CxA_2007 and CxA_2008 sheets report the QTL analyses in the CxA population for the years 2007 and 2008, respectively. WxBy_2010 and WxBy_2011 sheets report the QTL analyses in the WxBy population for the years 2010 and 2011, respectively. Position: map position in cM; Locus: markers IDs; LOD: LOD score; #iter.: the number of iteration needed to reach the tolerance criterium; mu_A, mu_H, and mu_B: estimated means of the distribution of the quantitative trait associated with the a-, h-, and b-genotypes; variance: the residual variance after fitting the QTL;%Expl.: percentage of the variance explained by the QTL; Additive: the estimated additive effect; Dominance: the estimated dominance effect; GIC: genotypic information coefficient. Click here for file

Additional file 3 **Details of the primers used with the Sequenom platform for the SNP genotyping of the CxA F**_**2 **_**population.** Click here for file

Additional file 4 Sequences of the primers used for the Sanger screening of the two NAC candidate genes and their upstream and downstream regions. Click here for file
